# SARS-CoV-2 in Malawi: Are we sacrificing the Youth in sub-Saharan Africa?

**DOI:** 10.7189/jogh.10.020336

**Published:** 2020-12

**Authors:** Biplap Nandi, Andreas Schultz, Minke HW Huibers, Amos Msekandiana, Msandeni Chiume-Kayuni

**Affiliations:** 1 Baylor College of Medicine, Lilongwe, Malawi; 2 College of Medicine, Department of Paediatrics, University of Malawi, Lilongwe, Malawi; 3 Kamuzu Central Hospital, Lilongwe, Malawi

Countries vary widely in terms of their capacity to prevent, detect, and respond to infectious disease outbreaks [1]. Living and working in Malawi, a United Nations (UN)-classified least developed country (LDC), we urge that control measures need to be adapted to the respective population and health care system. Measures intended to flatten the curve, presuppose spare capacity or ability to create it. Already overwhelmed health care systems in LDCs do not have such spare capacity.

In response to the SARS-COV-2 threat Malawi has closed schools and universities. As a result, pupils risk losing their only good meal a day, shelter from household violence and stipends, delaying graduation and their first job in life. Moreover, Malawi blood transfusion service depends on schools, colleges, places of worship, and workplaces [2]. Decreased blood stocks will increase preventable mortality. After Ebola, loss of family income, inflation and reduced access to health services lead to increased malnutrition and a rise in under five morbidity and mortality.

One of Africa’s biggest children’s departments, a 399-bed paediatric unit with up to 24 000 admissions per year, serving a population of five million is situated in central Malawi. Institutional responses and security measures in the light of SARS-COV-2 have resulted in loss of national and international medical staff at this institution, now facing a 66% decrease in consultants and 60% loss in non-consultant capacity. Established hospital partnerships struggle to meet their human resource commitments in a time they are needed more than ever and restoration of cooperation is not happening for an unforeseeable period of time.

Paediatric bed occupancy is reduced by 75% and there is a fear that these children might die at home from preventable causes or will present at advanced stages of their disease in the months to come. Elective operations, referrals and clinic visits have been minimised, delaying treatment follow up and drug supply. Malawi has a population of 67% below the age of 25 years and only 3% above the age of 65 [3]. Without yet knowing the effect of SARS-COV-2 on an underserved, malnourished and immuno-compromised youth, we still must consider the adverse effects of the current measures.

**Figure:**
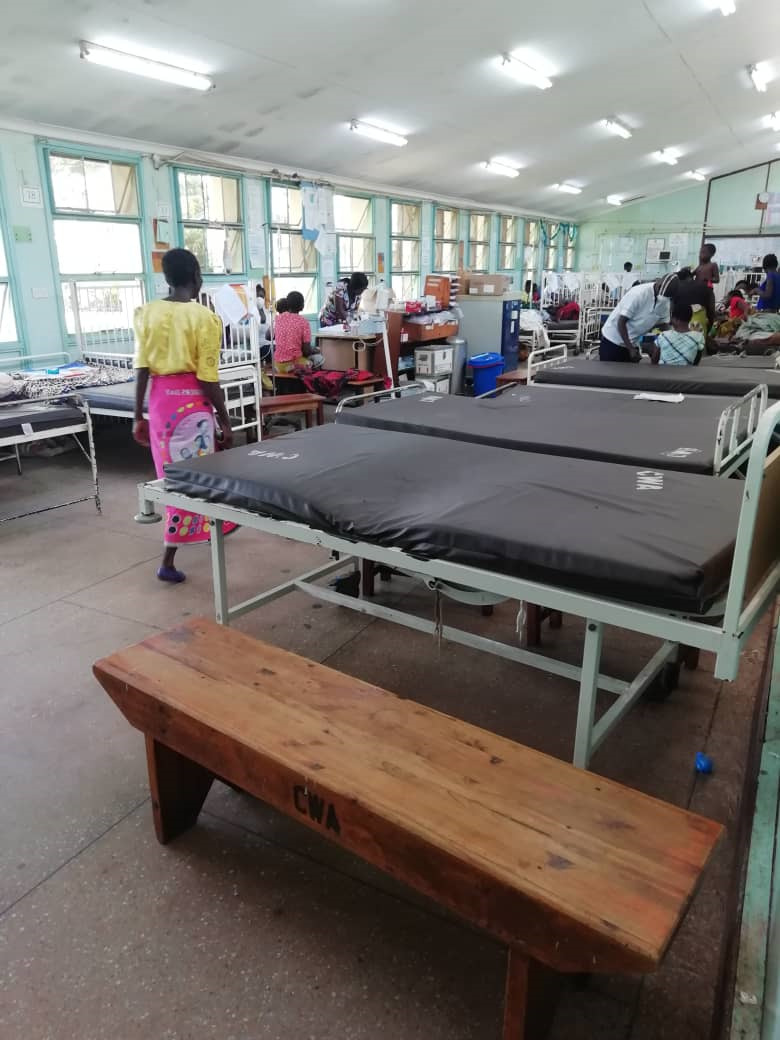
Photo: 50% of regular bed occupancy in emergency care (from Dr Biplap Nandi and Dr Andreas Schultz’s own collection, used with permission).

At the date of submission, 100 days have passed since detection of the first case and 86 days passed since closure of all schools nationwide. Without strict containment measures in Malawi, there have been 379 cases detected and 4 deaths attributable to the epidemic [4]. In anticipation of a yet unwitnessed influx of patients, paediatric department bed occupancy is at most 50% of the monthly median and services remain grossly reduced, including measures against HIV, Tb and malaria. Community interventions as well as primary and secondary health care are equally affected, not to mention the anticipated socio-economic cost for individual families.

Whilst such adverse effects can be mitigated in high-income countries with social security systems, injections of liquidity and ability to increase medical capacity, many LDCs do not have these safety nets. Risk benefit analyses are therefore different in highly vulnerable populations and they need to be adapted to the specific course of the epidemic in the country. Given the fact that especially African countries experience very different patterns of the global pandemic, we urge to be more pragmatic, context-specific and to consider the adverse effect of measures in place. Otherwise, we may counteract the intended outcomes and put our youth at even greater risk with regard to health, education and a prosperous future.
